# Life Satisfaction and the Relationship between Mild Cognitive Impairment and Disability Incidence: An Observational Prospective Cohort Study

**DOI:** 10.3390/ijerph18126595

**Published:** 2021-06-19

**Authors:** Osamu Katayama, Sangyoon Lee, Seongryu Bae, Keitaro Makino, Ippei Chiba, Kenji Harada, Yohei Shinkai, Hiroyuki Shimada

**Affiliations:** 1 Department of Preventive Gerontology, Center for Gerontology and Social Science, National Center for Geriatrics and Gerontology, 7-430 Morioka-cho, Obu 474-8511, Japan; sylee@ncgg.go.jp (S.L.); bae-sr@ncgg.go.jp (S.B.); kmakino@ncgg.go.jp (K.M.); ichiba@ncgg.go.jp (I.C.); harada-k@ncgg.go.jp (K.H.); yshinkai@ncgg.go.jp (Y.S.); shimada@ncgg.go.jp (H.S.); 2 Japan Society for the Promotion of Science, Tokyo 102-0083, Japan

**Keywords:** life satisfaction, mild cognitive impairment, disability

## Abstract

The relationship between the incidence of disability and cognitive function has been clarified, but whether life satisfaction is related to this relationship is unclear. Therefore, the purpose of this study was to clarify whether life satisfaction is related to the relationship between the incidence of disability and mild cognitive impairment. We included 2563 older adults from the National Center for Geriatrics and Gerontology–Study of Geriatric Syndromes. Baseline measurements included cognitive, life satisfaction, and demographic characteristics. Life satisfaction was measured using the Life Satisfaction Scale, which was stratified into three levels based on the score: lower, moderate, and higher. Associations between disability incidence and mild cognitive impairment were examined for each group according to life satisfaction, and monthly assessment for disability was monitored through long-term care insurance certification for at least 2 years from the baseline. At a 35.5-month mean follow-up, 150 participants had developed a disability. The potential confounding factors adjusted hazard for incidence of disability in the group with lower life satisfaction was 1.88 (CI: 1.05–3.35; *p* = 0.034) for mild cognitive impairment. Mild cognitive impairment was associated with disability incidence, and the effect was more pronounced among older adults with lower life satisfaction.

## 1. Introduction

Japan is expected to have the highest percentage of older adults in the world, with 39.9% of the population aged 65 years or older by 2050 [1]. In developed countries facing population aging, including Japan, many older adults need nursing care [1,2]. Since the introduction of Japan’s Long-Term Care Insurance (LTCI) system in 2000, the number of older adults who need LTCI services has been increasing. Currently, about 6.4 million people are using the Japanese LTCI system [3]. Dementia, cerebrovascular disease, and age-related weaknesses have been identified as the main causes of disability in older Japanese adults of both sexes [3]. Many longitudinal studies reported that the incidence of disability was associated with motor cognitive risk (MCR) syndrome [4], cognitive frailty [5], and low instrumental activity of daily living level [6]. The common denominator between MCR syndrome and cognitive frailty is that both definitions include mild cognitive decline and slow gait speed or comorbidity with physical frailty. Therefore, it is convincing that MCR syndrome and cognitive frailty are associated with disability incidence. However, when the prevalence of MCR syndrome and cognitive frailty was compared with mild cognitive impairment (MCI), the prevalence of MCI was found to be higher. Since the rate of dementia is increasing year by year as a major factor in the incidence of disability among older Japanese adults, it is important to focus on MCI, which is an intermediate state of Alzheimer’s disease (AD).

The increase of aging populations worldwide has been accompanied by an increase in the prevalence of dementia and AD [7], which has caused health, economic, and social burdens [8,9]. A 2014 study reported that there would be 46.8 million people with dementia worldwide by 2015 and that this number is projected to double every 20 years, reaching 74.7 million in 2030 and 131.5 million in 2050 [10]. Thus, reducing the incidence of dementia is a global necessity. MCI is the intermediate state of cognitive function between the changes seen in aging, dementia, and often AD [11]. This is concerning, given that up to 20% of people over the age of 65 years experience MCI [12,13]. To prevent dementia, the Japanese government is making efforts to provide early detection and early response to people with MCI and dementia [14]. The rationale for this is that in previous MCI longitudinal studies, many MCI cases did not develop dementia but reverted to normal cognition [15].

In a study conducted by Kim, Choe, and Lee [16], participants who fell in the dementia high-risk group showed significantly more negative results in terms of activities of daily living, depression, loneliness, social support, and life satisfaction than did participants in the low-risk group. In a longitudinal study of older adults without MCI or dementia, it was found that low life satisfaction was associated with the incidence of MCI and dementia [17,18]. Life satisfaction has been shown to be associated with suicide, mortality, apathy, and mental disorders (i.e., depression) [19]. Social and productive leisure participation in religious activities, social gatherings, and volunteering have been significantly related to the quality of life in older adults. Moreover, frequent participation in travel and cultural activities outside the home is positively related to life satisfaction [20]. Physical activity has been significantly related to life satisfaction and happiness in young, middle-aged, and older adults [21,22]. An individual with a more active lifestyle is less likely to develop cognitive decline and dementia than a less active individual [23,24,25,26]. Since the start of the COVID-19 pandemic in February 2020, opportunities for face-to-face interactions have been decreasing in Japan [27]. Before the COVID-19 pandemic, previous studies reported that one in four older adults were socially isolated and more than 40% experienced loneliness [28]. Decades of observational studies have demonstrated the long-term negative health outcomes of social isolation and loneliness [29,30]. The COVID-19 crisis has exacerbated these challenges, with worsening social isolation and loneliness among those who live alone or are frail and even declines in the well-being of older adults with previously active or healthy social lives [28]. Based on the results of these previous studies, the well-being of older adults is an important objective for both economic and health policymakers [31].

It is clear that there is a relationship between disability and cognitive function, as dementia has been the highest factor in the incidence of disability among older Japanese adults. However, there is a lack of research in the existing literature on whether life satisfaction influences this relationship. Therefore, the purpose of this study was to clarify whether life satisfaction is related to the relationship between the incidence of disability and MCI. We hypothesized a greater increase in the risk of disability by MCI among individuals with lower life satisfaction than among those with higher life satisfaction.

## 2. Materials and Methods

### 2.1. Design, Setting, and Participants

This research comprised an observational prospective cohort study of adults enrolled in a population-based cohort study, which is part of the National Center for Geriatrics and Gerontology–Study of Geriatric Syndromes (NCGG–SGS). The NCGG–SGS is a cohort study with the primary goal of establishing a screening system for geriatric syndromes and validating evidence-based interventions for preventing these syndromes [32].

The original plan of our study was to target the older population of 65 years and above. Takahama City provided full cooperation for this study, but requested us to include people aged 60 years and older; therefore, we included participants who resided in Takahama City and who were aged 60 years or older at the time of the study (September 2015 to February 2017). Takahama City provided us with the address information of residents aged over 60 years. We sent invitation letters inviting 9716 participants who were aged 60 years or older, lived in Takahama City, were not hospitalized, were not in residential care, were not certified by the LTCI system as having a functional disability, or were not participating in another study. All participants provided written informed consent at the study site prior to participating in the study. The study protocol was approved by the Ethics Committee of the National Center for Geriatrics and Gerontology (No. 1440). A total of 4167 community-dwelling older adults participated in baseline assessments, which included face-to-face interviews and physical and cognitive function evaluations. We excluded the following participants from the statistical analysis. (1) We excluded those aged less than 65 years (*n* = 777). This is because the Japanese public LTCI system is available to those aged 65 years and over when they require nursing care regardless of the cause, and to those aged 40–64 years when they require nursing care for specific diseases, such as terminal cancer and rheumatoid arthritis. Since the number of people aged 40–64 years who are certified as needing nursing care and their percentage of the insured population is as small as 0.4% [33], those under 65 years were excluded from the analysis in this study. (2) We excluded those with health problems (dementia, Parkinson’s disease, and stroke) (*n* = 240), based on information obtained by a qualified nurse who interviewed the participants face-to-face. (3) We excluded those who needed support or care—as certified by the Japanese public LTCI system—due to disability (*n* = 75). (4) We excluded those with disabilities affecting basic activities of daily living (ADL) (*n* = 5). (5) We excluded those with global cognitive decline at the baseline assessment (*n* = 235). Finally, (6) we excluded responses with missing variables of exclusion criteria (*n* = 272). Of the initial 4167 participants, 1604 were excluded based on these criteria. The study participants were followed from September 2015 to February 2019, with a mean follow-up at 35.5 months (SD: 5.7 months).

### 2.2. Measurement of Life Satisfaction

Life satisfaction was assessed using the Life Satisfaction Scale (LSS) [34] which included 13 items across five domains: (1) health, (2) community environment, (3) interpersonal relationships, (4) society, and (5) social roles. Health satisfaction was measured using two global questions: (1) “How satisfied are you with your mental health?” and (2) “How satisfied are you with your physical health?” Community environment satisfaction was measured using responses to three items: (3) “How satisfied are you with your housing?” (4) “How satisfied are you with your community environment or neighborhood?” and (5) “How satisfied are you with your household finances?” Interpersonal relationship satisfaction was measured as follows: (6) “How satisfied are you with your relationships with family members?” (7) “How satisfied are you with your relationships with friends?” and (8) “How satisfied are you with your relationships with neighbors?” Satisfaction with Japanese society was measured using responses to two items: (9) “How satisfied are you with Japanese social security, such as pensions and insurance?” and (10) “How satisfied are you with Japanese politics?” Satisfaction with social roles was measured using responses to the following three questions: (11) “How satisfied are you with your social role?” (12) “How satisfied are you with your accomplishments?” and (13) “How satisfied are you with the amount of free time you have for yourself outside of work or household chores?” The response options for all the questions were: 1 = poor, 2 = not very good, 3 = good, and 4 = excellent. For the analysis, the total satisfaction score was calculated by summing the individual scores (range = 13–52), with higher scores indicating higher overall satisfaction. The scale’s internal consistency validity was high [34]. Additionally, we created and operationally defined three categories to test for differences in the level of life satisfaction status related to the relationship between the incidence of disability and MCI. We divided the participants into three groups: those with lower life satisfaction (range = 13–37 scores), moderate satisfaction (range = 38–41 scores), and higher satisfaction (range = 42–52 scores), based on the distribution of total scores.

### 2.3. Measurement of Cognitive Function

For cognitive screening, the National Center for Geriatrics and Gerontology–Functional Assessment Tool (NCGG–FAT), which is an iPad application, was employed [35]. The tool comprises four domains: (1) memory (word list memory—I [immediate recognition] and word list memory—II [delayed recall]); (2) attention (a tablet version of the Trail Making Test—Part A); (3) executive function (a tablet version of the Trail Making Test—Part B), and (4) processing speed (a tablet version of the Symbol Digit Substitution Test). The tool has high test–retest reliability, moderate-to-high criterion-related validity [35], and predictive validity [36] among community-dwelling older adults. Cognitive assessments were conducted by the staff trained by the authors. Potential participants with MCI were identified after reviewing the available clinical, neuropsychological, and laboratory data at meetings involving study neurologists and neuropsychologists, as described in a previous study [37]. MCI participants were independently assessed using the NCGG–FAT, which has two memory tasks, attention and executive function tests, and a processing speed task. Using established criteria [38], MCI was diagnosed in individuals who exhibited cognitive impairment but were functionally independent in terms of basic daily life activities. For all cognitive tests, the established standardized thresholds were used in each corresponding domain to define impairment in population-based cohorts comprising community-dwelling older adults (scores of more than 1.5, standard deviations that specified age and educational means) [37,39]. Global cognitive function was measured using the Mini-Mental State Examination (MMSE) [40]. Participants whose cognitive test scores were all more than 1.5 standard deviation units above the mean were categorized into the normal cognition (NC) group.

### 2.4. Disability Determination

Participants were tracked monthly for a new incidence of LTCI certification, as recorded by the Japanese LTCI system and measured by the municipal government. The LTCI system classifies a person in “Support Level 1 or 2” to indicate a need for assistance to support ADLs, or in “Care Levels 1 through 5” to indicate a need for continuous care [41]. In this study, disability was defined as any LTCI certification level, and we defined disability onset as the point at which a participant received LTCI certification.

### 2.5. Potential Confounding Factors

Potential confounders of disability incidence included demographic variables, chronic diseases, psychological factors, and metabolic parameters associated with disability in older adults [6,42,43,44]. Therefore, our model included the following covariates: age at enrollment, sex, medication use, presence of chronic diseases, number of years of education, grip strength, MMSE [40] score, 15-item Geriatric Depression Scale (GDS) score [45], body mass index (BMI), total serum protein, albumin, and Walk Score. The presence of the following self-reported chronic diseases were also included among the covariates: heart disease, diabetes, hyperlipidemia, and spinal diseases. Grip strength was measured using a Smedley-type handheld dynamometer (GRIP-D; Takei Scientific Instruments Co., Ltd., Niigata, Japan) under strictly standardized conditions in which the same device was used to avoid inter-observer and inter-device variability. We recorded one measurement of grip strength of each participant’s dominant hand, with participants in a standing position and with their elbows extended. We utilized the Walk Score™ (Front Seat Management, Limited Liability Company [LLC], Seattle, WA, USA) [46], a publicly available website found to be valid and reliable for estimating accessibility to amenities within a comfortable walking distance [47]. The Walk Score uses data provided by the Google™ AJAX Search application program interface [48], along with a geography-based algorithm to identify nearby amenities, and calculates a “walkability” score [46] based on the distance to amenities. Low walkability has been associated with cognitive decline and disability [6,49]. In this study, the Walk Score was calculated for all participants using their home addresses. Blood samples, taken to check total serum protein or albumin, were collected at least 4 h after meals and were analyzed using standard laboratory techniques. Potential confounders of life satisfaction included age, sex, medication use, grip strength, MMSE, GDS, Walk Score, loneliness, self-rated health, and physical and social activities) [50,51]. Loneliness was measured using a single question from a three-item loneliness scale [52], “I feel isolated from others.” Self-rated health was measured using a single question, “In general, how would you rate your health?” with the following response options: good, rather good, poor, and very poor [53]. Physical activity was assessed at baseline by asking participants about their participation in the following activities during the past year: walking, cycling, jogging, swimming, muscle training, yoga, gymnastics, dancing, hiking, playing golf, playing grand golf, or ball exercise. Exercise frequency was assessed by participants as never, once a month or less, several times a month, 1–2 times per week, 3–6 times per week, and every day [54]. Individuals in this study, except for those who did not participate at least once a week, were classified as regular participants. Social activity was assessed at baseline by asking participants about their participation in the following activities involving two or more people during the past year: being an officer of a senior club or neighborhood association, attending a regional event, engaging in environmental beautification activities, teaching, supporting activity, working, singing karaoke, dining out, partying with friends, shopping with friends, talking to friends (including phone calls), attending an event or concert, or traveling. The frequency of participation in social activities was also assessed by participants as follows: never, once a month or less, several times a month, 1–2 times per week, 3–6 times per week, and every day [54]. Individuals in this study, except for those who did not participate at least once a year, were classified as regular participants.

### 2.6. Statistical Analysis

One-way analysis of variance and Pearson’s chi-square tests were used to compare variables among groups of participants who were disability-free, developed a disability, and died or relocated. Unpaired *t*-tests were used to compare variables among participants with NC and MCI. Adjusted standardized residuals > 1.96 indicated *p* < 0.05. We calculated cumulative incident disability during follow-ups for each of the two abovementioned cognitive status groups using Kaplan–Meier curves. Intergroup differences were estimated using the log-rank test. Crude and adjusted Cox proportional hazard models were constructed to calculate hazard ratios (HRs) with 95% confidence intervals (CIs) for incident disability risk. Model 1 is a crude model. Model 2 is adjusted for age and sex. The factors associated with the incidence of disability among older Japanese have been shown to differ by age and gender [55,56]. In previous studies examining factors associated with disability incidence in older Japanese, models that adjust for age and gender have been used in many cases [42,43,57,58]. Model 3 is adjusted for the covariates in Model 2 and years of education, heart disease, diabetes, hyperlipidemia, spinal diseases, BMI, total serum protein, albumin, medication, MMSE score, GDS score, grip strength, and Walk Score. Given the significant effect of life satisfaction on disability, we performed analyses by applying Cox proportional hazard models to the lower, moderate, and higher life satisfaction groups separately. Finally, multinomial logistic regression analysis was conducted as a sub-analysis to clarify the factors related to the level of life satisfaction based on cognitive status. Data are presented as odds ratios (ORs) with 95% confidence intervals (CIs). The significance level was set at *p* < 0.05. All analyses were performed using IBM SPSS version 25.0 (IBM, Tokyo, Japan).

## 3. Results

The final analysis included data from 2563 older adults (1476 women; mean age 72.6 years, standard deviation [SD] 5.7 years, age range 65–96 years). Of the 2563 participants, 2346, 150, and 67 participants remained disability-free, developed a disability, and died or relocated, respectively. Table 1 shows the baseline characteristics of the study participants who remained disability-free, developed a disability, and died or relocated from the study. At baseline, participants who had died or relocated were older, took more medication, had less education, higher GDS scores, and lower albumin levels than those who were followed up and remained disability-free (*p* < 0.001). The mean number of LSS scores for participants who died or relocated was 39.3 (SD: 6.0), compared with 39.3 (SD: 5.3) for those who remained in the study. Of the 67 participants who died or relocated, 31 (46.3%) had LSS scores lower than the mean.

**Figure 1:**
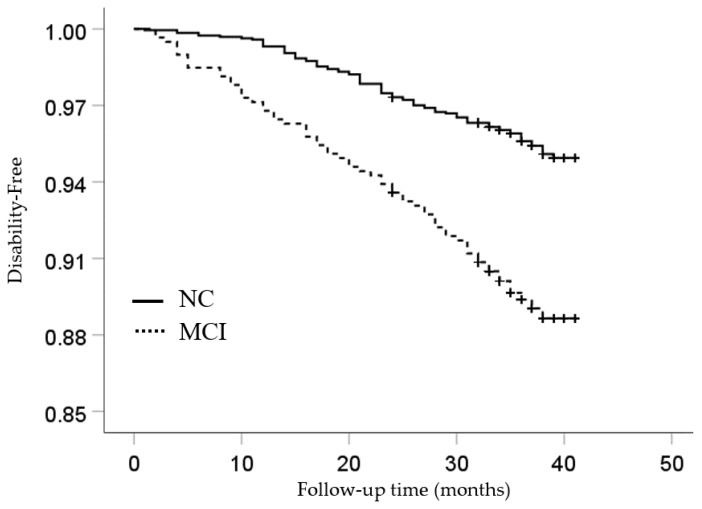
Kaplan–Meier survival estimates by cognitive status groups. Note: Those participants who had NC had a higher probability of being disability-free than those who had MCI.

Table 2 shows the baseline characteristics according to cognitive status. The incidence of disability was 14.9/1000 person-years for those who had NC, compared with 35.8/1000 person-years for individuals with MCI. Figure 1 shows the probabilities of being disability-free by cognitive status among participants in each life satisfaction group. Survival analyses with Kaplan–Meier log-rank test showed that participants who had NC had a higher probability of being disability-free than those who had MCI (*p* < 0.001). The potential confounder-adjusted disability HR for participants in the MCI group was 1.59 (CI: 1.13–2.25; *p* = 0.008). The interaction between cognitive status and life satisfaction was statistically significant (*p* = 0.003).

The potential confounding factors adjusted HRs for incidence of disability in the group with lower life satisfaction were 1.88 (CI: 1.05–3.35; *p* = 0.034) for MCI. However, in the groups with moderate and highest life satisfaction, MCI was 1.79 (CI: 0.89–3.57; *p* = 1.101) and 1.28 (CI: 0.71–2.29; *p* = 0.414), respectively (Table 3).

The sub-analyses of the factors related to the level of life satisfaction by cognitive status (Table 4), shows the ORs and 95% CIs estimated by adjusted multinomial logistic regression analyses for each cognitive status, with the level of life satisfaction as the dependent variable (using the higher life satisfaction group as a reference) and factors related to life satisfaction as the independent variable. After adjusting for potential confounding factors (i.e., demographic variables, psychological factors, and activities), in the lower life satisfaction group, the NC group was found to be independently associated with age, sex, GDS, loneliness, self-rated health, and participation in social activities; the MCI group was found to be independently associated with age, GDS, loneliness, and self-rated health (all *p* < 0.05).

## 4. Discussion

In this observational prospective cohort study of adults enrolled in a population-based cohort study, we found an increased incidence rate of disability for individuals with MCI (35.8/1000 person-years) compared with those who had NC (14.9/1000 person-years). The potential confounding factors adjusted HRs for incidence of disability in the group was 1.59 (CI: 1.13–2.25; *p* = 0.008) for MCI; this corresponds to a 59% rise in the disability risk of participants with MCI. MCI seemed to be associated with the greatest risk increase in participants who had lower levels of life satisfaction at baseline.

As an innovative feature of our study, we found a potentially important association between cognitive status and life satisfaction in relation to the incidence of disability. There was a greater risk of disability based on cognitive status among individuals with lower life satisfaction than among those with higher life satisfaction. Lower life satisfaction was associated with an increased disability risk among individuals with MCI; however, this increased risk diminished among individuals who had NC. Our findings suggest that one of the ways in which life satisfaction may reduce the risk for disability is by modulating the relationship between MCI and disability—an area worthy of additional investigation. We recognize that further longitudinal studies are needed to determine the role of life satisfaction. We are very interested in whether life satisfaction plays a role in modulating the relationship between MCI and disability, and we have already started a longitudinal study. Previous studies have reported that older adults with cognitive impairment have lower levels of life satisfaction [50]. Furthermore, in a longitudinal study of older adults without MCI or dementia, it was found that low life satisfaction was associated with the incidence of MCI and dementia [17,18]; therefore, the risk of developing disability might have been reduced in the group that had high life satisfaction, even in those with MCI. These findings suggest that increasing life satisfaction is effective in preventing disability in older adults with MCI. Recently, the COVID-19 pandemic has even led to declines in the well-being of older adults with previously active or healthy social lives [28]. Therefore, it may be important to develop effective strategies, not only to help MCI but also to provide care and support to help older adults maintain a high level of life satisfaction.

The results of the sub-analysis examining factors associated with lower life satisfaction by cognitive status revealed that older adults with NC and MCI were all strongly associated with demographic variables and psychological factors. A previous study of older adults with reduced self-care capacity showed that factors significantly predicting low life satisfaction were poor overall self-reported health, poor financial resources in relation to needs, severe or totally impaired self-care capacity, feeling lonely sometimes or quite often, and feeling worried very often. Poor self-rated health and poor financial resources in relation to needs were most strongly related to low life satisfaction, while age, gender, living conditions, and participation in physical activities did not explain low life satisfaction [51]. By contrast, in a previous study examining cognitive impairment and life satisfaction in older adults, those with cognitive impairment had lower life satisfaction than those with NC, but the effect was relatively small [50]. Although the subjects in this study also had MCI, they were older adults living independently in the community, so there was no clear difference in the factors related to the decline in life satisfaction based on cognitive status.

The results of this study suggest the importance of assessing the life satisfaction of clients under care and incorporating the assessment into care and support plans when considering disability prevention for older adults with cognitive decline. The strengths of this study include its large sample size and life satisfaction assessment. To the best of our knowledge, this is the first study to evaluate cognitive status and level of life satisfaction to examine the relationship between life satisfaction and disability incidence. Dementia accounts for a large percentage of disability incidence in the elderly Japanese population [3]. In a longitudinal study of older adults without MCI or dementia, it was found that low life satisfaction was associated with the incidence of MCI and dementia [17,18]. The results of this study suggest that life satisfaction is related to the relationship between MCI and disability incidence. In the context of the COVID-19 pandemic, considering measures to maintain and improve the life satisfaction of older adults during the pandemic may prevent the incidence of disability in the future. To achieve this, clinicians, researchers, and the government need to work together.

This study also has some limitations. First, we sent invitation letters to individuals (*n* = 9716) who were aged 60 years or older, lived in Takahama City, were not hospitalized, were not in residential care, were not certified by the LTCI system as having a functional disability, or were not participating in another study. However, the participants in our study were only older adults who had access to health checkups from their homes, which implies that the sample excluded people with various other conditions. Second, we did not address other covariates related to economic status and well-being variables that could also affect life satisfaction; therefore, future studies should include such variables. Third, we were not able to clarify whether life satisfaction even has a role in moderates the relationship between MCI and disability. We are very interested in this topic and have already started a longitudinal study. Fourth, we had fewer participants who were older adults with MCI. Thus, we might have underestimated the incidence of disability in older adults with MCI. Future studies should investigate the association between phenotype in MCI and incidence disability in more detail. Finally, we tracked the monthly incidence of new LTCI certifications as recorded by the Japanese LTCI system and measured by the municipality. However, information on the causes of disability at the follow-up was not available. Thus, we did not know whether the older adults with MCI during the follow-up period had developed dementia or other diseases.

## 5. Conclusions

MCI was associated with disability incidence, with a more pronounced effect among older adults who had a low level of life satisfaction. Given the increasingly high prevalence of disability and its strong association with numerous adverse health outcomes, clinicians can focus more on considering older adults’ cognitive status and life satisfaction in their day-to-day practice in adult-centered care. This, in turn, may lead to better outcomes in primary disability prevention.

## Figures and Tables

**Table 1:** Baseline characteristics of study participants by follow-up status.

Variable	Total	Participants Free of Disability	Participants with Disability	Participants Who Died or Relocated	*p*-Value	Post Hoc
	(*n* = 2563)	(*n* = 2346)	(*n* = 150)	(*n* = 67)		
Mean age at baseline, y	72.6 ± 5.7	72.1 ± 5.4	79.0 ± 6.0	75.7 ± 6.5	<0.001 *	Free < Died or relocated < Disability
Sex, Female (%)	1476 (57.6)	1350 (57.5)	94 (62.7)	32 (47.8)	0.120 ^†^	
Medication use, *n*	2.9 ± 2.6	2.8 ± 2.5	3.8 ± 2.5	3.9 ± 3.1	<0.001 *	Free < Disability, Died or relocated
Chronic disease						
Heart disease, no (%)	2145 (83.7)	1963 (83.7)	127 (84.7)	55 (82.1)	0.891 ^†^	
Diabetes, no (%)	2200 (85.8)	2019 (86.1)	125 (83.3)	56 (83.6)	0.562 ^†^	
Hyperlipidemia, no (%)	1740 (67.9)	1583 (67.5)	107 (71.3)	50 (74.6)	0.306 ^†^	
Spinal diseases, no (%)	2061 (80.5)	1891 (80.7)	117 (78.0)	53 (79.1)	0.689 ^†^	
Years of education, y	11.2 ± 2.3	11.2 ± 2.3	10.4 ± 2.4	10.5 ± 2.4	<0.001 *	Disability, Died or relocated < Free
Physical function						
Grip strength, kg	27.4 ± 7.6	27.7 ± 7.6	24.0 ± 6.8	26.6 ± 7.2	<0.001 *	Disability < Died or relocated, Free
Cognitive function						
MMSE, Score	27.6 ± 2.0	27.7 ± 2.0	26.9 ± 1.8	27.1 ± 1.9	<0.001 *	Disability < Free
GDS, Score	2.8 ± 2.5	2.7 ± 2.5	3.8 ± 2.9	3.6 ± 3.1	<0.001 *	Free < Disability, Died or relocated
BMI, kg/m^2^	23.5 ± 3.3	23.5 ± 3.3	23.1 ± 3.9	23.6 ± 4.2	0.404 *	
Metabolic parameters						
Total serum protein, g/dL	7.4 ± 0.4	7.5 ± 0.4	7.4 ± 0.5	7.4 ± 0.5	0.491 *	
Albumin, g/dL	4.4 ± 0.3	4.5 ± 0.3	4.4 ± 0.4	4.4 ± 0.3	<0.001 *	Disability, Died or relocated < Free
Walk Score	68.0 ± 11.6	68.1 ± 11.5	67.3 ± 12.3	68 ± 12.6	0.727 *	
LSS, Score	39.3 ± 5.3	39.2 ± 5.2	40.1 ± 5.6	39.3 ± 6.0	0.160 *	

* *p*-values reported from one-way ANOVA. Significant difference obtained by Tukey post-hoc test. ^†^ *p*-values obtained by Pearson’s chi-square test. MMSE, Mini-Mental State Examination; GDS, 15-item Geriatric Depression Scale; BMI, body mass index; LSS, life satisfaction scale; *n*, number; y, years.

**Table 2:** Baseline characteristics of study participants by cognitive status.

Variable	NC	MCI	*p*-Value
	(*n* = 1947)	(*n* = 616)	
Mean age at baseline, y	72.2 ± 5.6	73.7 ± 6.0	<0.001 *
Sex, Female (%)	1132 (58.1)	344 (55.8)	0.315 ^†^
Medication use, *n*	2.8 ± 2.5	3.1 ± 2.8	0.004 *
Chronic disease			
Heart disease, no (%)	1629 (83.7)	516 (83.8)	0.954 ^†^
Diabetes, no (%)	1688 (86.7)	512 (83.1)	0.026 ^†^
Hyperlipidemia, no (%)	1316 (67.6)	424 (68.8)	0.577 ^†^
Spinal diseases, no (%)	1569 (80.7)	492 (80.0)	0.715 ^†^
Years of education, y	11.3 ± 2.4	10.9 ± 2.2	<0.001 *
Physical function			
Grip strength, kg	27.8 ± 7.6	26.2 ± 7.3	<0.001 *
Cognitive function			
MMSE, Score	27.8 ± 1.9	26.9 ± 1.9	<0.001 *
GDS, Score	2.7 ± 2.5	3.0 ± 2.7	0.015 *
BMI, kg/m^2^	23.5 ± 3.2	23.4 ± 3.7	0.374 *
Metabolic parameters			
Total serum protein, g/dL	7.4 ± 0.4	7.5 ± 0.4	0.665 *
Albumin, g/dL	4.5 ± 0.3	4.4 ± 0.3	<0.001 *
Walk Score	67.8 ± 11.7	68.6 ± 11.4	0.132 *
LSS, Score	39.3 ± 5.2	39.2 ± 5.4	0.794 *

* *p*-values reported from unpaired *t*-test. ^†^ *p*-values obtained by Pearson’s chi-square test. MMSE, Mini-Mental State Examination; GDS, 15-item Geriatric Depression Scale; BMI, body mass index; LSS, life satisfaction scale; *n*, number; y, years.

**Table 3:** Cox proportional hazard models of the relationships between cognitive status and incident disability in each life satisfaction group.

**All Participants**	**Number of Participants**	**Incident Disability Rate**	**Model 1**			**Model 2**			**Model 3**		
			**HR**	**95% CI**	***p***	**HR**	**95% CI**	***p***	**HR**	**95% CI**	***p***
Normal cognition	1904	87 (4.6%)	1.00			1.00			1.00		
Mild cognitive impairment	592	63 (10.6%)	2.45	1.77–3.39	<0.001	1.87	1.35–2.60	< 0.001	1.59	1.13–2.25	0.008
**Lower Life Satisfaction Group** **(LSS = 13–37)**	**Number of Participants**	**Incident Disability Rate**	**Model 1**			**Model 2**			**Model 3**		
			**HR**	**95% CI**	***p***	**HR**	**95% CI**	***p***	**HR**	**95% CI**	***p***
Normal cognition	692	30 (4.3%)	1.00			1.00			1.00		
Mild cognitive impairment	227	24 (10.6%)	2.60	1.52–4.45	<0.001	2.07	1.20–3.55	0.008	1.88	1.05–3.35	0.034
**Moderate Life Satisfaction Group** **(LSS = 38–41)**	**Number of Participants**	**Incident Disability Rate**	**Model 1**			**Model 2**			**Model 3**		
			**HR**	**95% CI**	***p***	**HR**	**95% CI**	***p***	**HR**	**95% CI**	***p***
Normal cognition	607	23 (3.8%)	1.00			1.00			1.00		
Mild cognitive impairment	188	16 (8.5%)	2.31	1.22–4.38	0.010	1.91	1.01–3.62	0.047	1.79	0.89–3.57	1.101
**Higher Life Satisfaction Group** **(LSS = 42–52)**	**Number of Participants**	**Incident Disability Rate**	**Model 1**			**Model 2**			**Model 3**		
			**HR**	**95% CI**	***p***	**HR**	**95% CI**	***p***	**HR**	**95% CI**	***p***
Normal cognition	605	34 (5.6%)	1.00			1.00			1.00		
Mild cognitive impairment	177	23 (13.0%)	2.45	1.44–4.16	0.001	1.68	0.97–2.90	0.064	1.28	0.71–2.29	0.414

Notes: Model 1 is a crude model. Model 2 is adjusted for age and sex. Model 3 is adjusted for the covariates in Model 2 and years of education, heart disease, diabetes, hyperlipidemia, spinal diseases, BMI, total serum protein, albumin, medication, MMSE score, GDS score, grip strength, and Walk Score. LSS: Life satisfaction scale, HR: Hazard ratio.

**Table 4:** Multinomial logistic regression analysis with cognitive status as the dependent variable by the level of life satisfaction.

	NC			MCI		
	OR	95% CI	*p*-Value	OR	95% CI	*p*-Value
**Moderate life satisfaction (LSS 38–41)**						
Age, y	0.96	0.94–0.98	<0.001	0.96	0.92–1.00	0.027
Sex						
Male (ref.)	1.00			1.00		
Female	0.71	0.56–0.90	0.005	1.32	0.86–2.04	0.210
Medication, *n*	1.06	1.01–1.12	0.027	1.03	0.95–1.12	0.502
Grip strength						
Male; ≥28 kg, female; ≥18 kg (ref.)	1.00			1.00		
Male; <28 kg, female; <18 kg	0.82	0.56–1.21	0.326	0.70	0.39–1.28	0.251
MMSE, score	0.96	0.90–1.02	0.216	1.10	0.98–1.23	0.096
GDS, score						
0–5 (ref.)	1.00			1.00		
6–15	1.79	0.99–3.25	0.054	1.39	0.60–3.21	0.439
Walk Score						
70–100 (ref.)	1.00			1.00		
0–69	0.88	0.70–1.11	0.284	1.31	0.86–1.99	0.204
Loneliness						
No (ref.)	1.00			1.00		
Yes	2.09	1.62–2.70	<0.001	2.25	1.40–3.63	0.001
Self-rated health						
Good (ref.)	1.00			1.00		
Poor	2.81	1.81–4.37	<0.001	1.66	0.89–3.09	0.113
Walking habit						
≥3 times/week (ref.)	1.00			1.00		
<3 times/week	0.98	0.91–1.06	0.664	0.88	0.77–1.01	0.066
Social activities						
5–12 (ref.)	1.00			1.00		
0–4	0.90	0.86–0.95	<0.001	1.04	0.96–1.13	0.353
**Lower life satisfaction (LSS 13–37)**						
Age, y	0.92	0.90–0.95	<0.001	0.92	0.88–0.95	<0.001
Sex						
Male (ref.)	1.00			1.00		
Female	0.56	0.43–0.72	<0.001	0.96	0.62–1.48	0.852
Medication, *n*	1.04	0.98–1.10	0.170	1.00	0.92–1.06	0.952
Grip strength						
Male; ≥28 kg, female; ≥18 kg (ref.)	1.00			1.00		
Male; <28 kg, female; <18 kg	0.87	0.58–1.29	0.481	1.47	0.84–2.56	0.177
MMSE, score	0.97	0.91–1.03	0.342	1.04	0.92–1.16	0.548
GDS, score						
0–5 (ref.)	1.00			1.00		
6–15	8.00	4.67–13.68	<0.001	4.66	2.23–9.71	<0.001
Walk Score						
70–100 (ref.)	1.00			1.00		
0–69	1.01	0.79–1.29	0.951	0.95	0.62–1.46	0.804
Loneliness						
No (ref.)	1.00			1.00		
Yes	3.26	2.51–4.24	<0.001	3.11	1.95–4.96	<0.001
Self-rated health						
Good (ref.)	1.00			1.00		
Poor	4.11	2.64–6.40	<0.001	2.60	1.42–4.74	0.002
Walking habit						
≥3 times/week (ref.)	1.00			1.00		
<3 times/week	0.92	0.85–1.00	0.053	0.98	0.85–1.12	0.722
Social activities						
5–12 (ref.)	1.00			1.00		
0–4	0.85	0.80–0.89	<0.001	0.94	0.86–1.03	0.169

MMSE, Mini-Mental State Examination; GDS, 15-item Geriatric Depression Scale; ref, reference.

## Data Availability

Not applicable.
